# Exploring the gut microbiota-microbial metabolites-targets regulatory network in metabolic dysfunction-associated steatotic liver disease

**DOI:** 10.3389/fmolb.2026.1764479

**Published:** 2026-02-09

**Authors:** Dapeng Yin, Wei Wu, Xiaojuan Wang, Junhua He, Yikun Zhu, Jin Li

**Affiliations:** 1 Department of Endocrinology and Metabolism, The Second Hospital of Shanxi Medical University, Taiyuan, Shanxi, China; 2 The Second Clinical Medical College of Shanxi Medical University, Taiyuan, Shanxi, China

**Keywords:** gut microbiota, lipid metabolism, MASLD, mendelian randomization, microbial metabolites

## Abstract

**Background:**

Metabolic dysfunction-associated steatotic liver disease (MASLD), which has emerged as a significant global public health concern. The regulatory role of gut microbiota and their metabolites in the pathogenesis of MASLD has attracted significant attention.

**Methods:**

Mendelian randomization (MR) analysis was performed to identify gut microbiota that were causally associated with MASLD. Evaluate the metabolites derived from the microbiota and predict their targets. Obtaining overlapping targets between metabolites and MASLD. The core targets were screened by protein-protein interaction (PPI) network, and functional enrichment analysis were performed. Binding interactions between metabolites and targets are validated through molecular docking. Meanwhile, the MASLD animal model was used to verify the expression differences of the core targets. Finally, the regulatory network of gut microbiota-metabolites-targets was constructed.

**Results:**

We identified 11 gut microbiota causally linked to MASLD by MR analysis. And 19 kinds of microbial metabolites were obtained. Enrichment analysis revealed that the intersection targets were concentrated cholesterol metabolism, PPAR and HIF-1 signaling pathways. Four core targets were identified, including ACE, HMGCR, PPARA and PPARG. Through molecular docking simulations, we predicted that there is potential stable binding affinity between the core targets and metabolites. Notably, a marked difference expression of key target genes was identified in the MASLD mice.

**Conclusion:**

Gut microbiota and microbial metabolites played an important role in the development of MASLD. This involved a multi-target and multi-pathway regulatory network, providing new evidence for the mechanism research and targeted intervention of MASLD.

## 1 Introduction

Metabolic dysfunction-associated steatotic liver disease (MASLD) is recognized as a chronic liver condition. Due to the growing occurrence of obesity, metabolic syndrome, type 2 diabetes, hyperlipidemia and other diseases worldwide, the incidence of MASLD has been progressively increasing. This has turned into a pressing public health issue internationally (Leca et al., 2024). The pathogenesis of MASLD is complex and usually involves the interaction of multiple factors such as insulin resistance, immune dysfunction, lipid metabolism disorder, mitochondrial dysfunction, intestinal flora imbalance, and activation of inflammatory signaling pathways (Buchynskyi et al., 2025). This eventually leads to lipid accumulation in the liver and damage to liver cells. Due to the complex pathogenesis of MASLD, current treatment methods are still limited. In light of this, there is a pressing need for comprehensive research into the underlying mechanisms of MASLD and the identification of novel therapeutic targets, which has become a significant scientific challenge in hepatology.

The gut microbiota and its metabolites are integral to the onset and progression of MASLD. Recent investigations have highlighted a strong association between the gut microbiota and the development of MASLD, as it can influence the host’s liver metabolism and systemic immune response through metabolic regulation and immune modulation. Specific microbial communities can directly affect the host’s metabolic function through their metabolic products, promoting fat deposition and causing liver damage. For instance, short-chain fatty acids (SCFAs) associated with microbial metabolites such as acetate, propionate, and butyrate not only have anti-inflammatory effects, but also improve insulin sensitivity, regulate lipid metabolism, and alleviate hepatic lipotoxicity (Guo et al., 2023). Moreover, dysbiosis of gut microbiota can weaken the intestinal barrier, permitting endogenous toxins like endotoxins to enter the bloodstream and activate systemic immune-inflammatory responses (Wang et al., 2025). This further exacerbates hepatic inflammation and lipotoxicity. However, the gut microbiota exhibits remarkable complexity and diversity, and its precise mechanisms remain incompletely understood. Therefore, thoroughly understanding how the gut microbiota and its metabolites affect MASLD at the molecular level may offer new prevention and treatment strategies.

Emerging bioinformatics techniques such as Mendelian randomization (MR) analysis and network pharmacology offer novel perspectives for investigating the pathogenesis of MASLD. Based on the biological characteristics of random allele allocation of single nucleotide polymorphisms (SNPs), MR analysis has proven effective in mitigating confounding factors (Ference et al., 2021). This helps to reveal the causal relationship between the gut microbiota and MASLD. Through network pharmacology analysis, key targets of microbial metabolites can be identified while simultaneously exploring potential biomarkers and intervention strategies (Yao et al., 2025). Although MR analysis provides a powerful tool for identifying causal associations, single-dimensional analysis is difficult to explore the mechanisms of multi-network and multi-biological level interactions behind MASLD. Therefore, by integrating MR analysis and network pharmacological analysis, a causal regulatory network from the microbiota to the target is constructed. This has become an urgent need to systematically clarify the pathogenesis of MASLD.

This research combined MR analysis and network pharmacology approaches to investigate the causal relationships and core targets of MASLD. Meanwhile, an animal model of MASLD was constructed to further verify the key role of the target. By constructing a network of gut microbiota - metabolites - targets, potential regulatory hierarchies and biomarkers were revealed. From the angle of combination of genetics and systems biology, this study MASLD treatment provides theory basis for the future.

## 2 Methods and materials

### 2.1 MR analysis

MiBioGen, an international consortium, aggregated genome-wide association studies (GWAS) data on gut microbiota. A total of 211 taxonomic units of intestinal microbiota were annotated from this dataset, covering 9 phyla, 16 classes, 20 orders, 35 families and 131 genera (Kurilshikov et al., 2021). The data of MASLD were obtained from the IEU Open GWAS database (https://gas.mrcieu.ac.uk/), and the dataset ebi-a-GCST90054782 with a large sample size and SNPs was selected as the outcome data. A total of 377,998 European participants were included in the data set, which consisted of 4,761 cases and 373,227 controls. MR analysis follows the following three assumptions: (1) Correlation assumption: instrumental variables (IVs) is closely related to exposure, but does not directly affect the outcome. (2) Independence assumption: IVs are not affected by confounding factors. (3) Exclusion hypothesis: The impact of IVs on the outcome is only through exposure. Guided by these three core assumptions, this study adopted a rigorous, multi-step process to select SNPs significantly associated with the target gut microbiota to serve as IVs. Firstly, in accordance with the correlation assumption, SNPs that were significantly associated with the gut microbiota were screened out using a threshold of *P* < 1 × 10^−5^ as the criterion (Xiang et al., 2023). By calculating the F statistic (calculation formula: F = β^2^/se^2^, where β is the allele effect value and se is the standard error), weak IVs with F < 10 were eliminated to ensure a stable and strong association between the IVs and the gut microbiota exposure. Subsequently, for the independence assumption, the linkage disequilibrium (LD) screening criteria (r^2^ < 0.001, SNP physical distance >10,000 kb) were used to eliminate the candidate SNPs that showed collinearity. At the same time, the LDtrait tool (https://ldlink.nih.gov/?tab=ldtrait) was utilized to comprehensively detect the association between the IVs and potential confounding factors (Lin et al., 2020). Finally, for the exclusionary assumption, SNPs with palindromic structures (i.e., alleles being A/T or G/C) were excluded. Subsequently, the directional horizontal pleiotropy was evaluated through the subsequent MR-Egger regression analysis. Finally, the SNPs that met the criteria were selected as the IVs for exposure and included in the subsequent analysis.

This research conducted a two-sample MR analysis with 211 gut microbiota as exposure and the outcome variable MASLD. It mainly included five analysis methods, namely inverse-variance weighted (IVW) test, MR-Egger, Weighted median, Simple mode and Weighted Mode. Take IVW as the main method (Xu et al., 2022). After MR analysis, sensitivity analysis was conducted through various methods to evaluate the reliability of the results. The heterogeneity among instrumental variables was evaluated using Cochran’s Q test, and a Q_pval <0.05 signifies its presence. Horizontal pleiotropy was tested using MR-PRESSO and MR-Egger regression intercepts. To assess the robustness of the causal estimates, a leave-one-out analysis was carried out. Finally, the gut microbiota with differences were obtained and visualized by scatter plot. *P* value <0.05 was viewed as statistically significant.

### 2.2 Identify the metabolites of the gut microbiota and their corresponding targets

Metabolites produced by the gut microbiota were discovered using the gutMGene database (https://bio-computing.hrbmu.edu.cn/gutmgene/#/Home) (Qi et al., 2025). Through the Pubchem database (https://pubchem.ncbi.nlm.nih.gov/) to obtain the metabolites SMILE formula. Metabolite target predictions were obtained using the SwissTargetPrediction database (https://www.swisstargetprediction.ch/?) and the Similarity ensemble approach (SEA) database (https://sea.bkslab.org/) (Daina et al., 2019). Ultimately, the intersection targets between the two databases were calculated using a Veen diagram.

### 2.3 Identification of metabolites and MASLD common targets

MASLD targets were sourced from GeneCards Database (https://www.genecards.org/), the Comparative Toxicogenomics Database (http://ctdbase.org/), and the OMIM database (https://www.omim.org/) (Safran et al., 2010; Davis et al., 2023; Amberger et al., 2019). Then overlapping targets between metabolites and MASLD were identified using Veen diagrams.

### 2.4 Construction of protein-protein interaction (PPI) networks and identification of hub genes

The STRING (https://cn.string-db.org/) platform was used to build PPI networks (Szklarczyk et al., 2023). We set the threshold for inclusion at a “medium confidence” level, which corresponds to an interaction score of 0.400. This ensured a high-confidence association. In Cytoscape (version3.10), we used five algorithms (Degree, EPC, MCC, Radiality, Stress) to identify the core targets in the network to ensure the reliability of the results.

### 2.5 Gene ontology (GO) and kyoto encyclopedia of genes and genomes (KEGG) enrichment analysis

DAVID (https://davidbioinformatics.nih.gov/) was a database that provides functional annotations by obtaining a large number of gene lists from high-throughput genomic studies (Dennis et al., 2003). We submitted overlapping targets to the DAVID database for functional annotation. GO enrichment includes three processes: Biological Process (BP), Cellular Component (CC) and Molecular Function (MF). KEGG enrichment analysis can be used to identify whether biological pathways are significantly expressed. The significantly enriched pathways were screened with *P* < 0.05 and FDR <0.05.

### 2.6 Identify potential interacting proteins of the core targets

To avoid the one-sidedness of the research results, we used the GeneMANIA database (https://genemania.org/) to conduct in-depth expansion of the core targets (Franz et al., 2018). The aim was to identify potential interacting proteins associated with these targets. Subsequently, we constructed a PPI network for the expanded target and conducted enrichment analyses in GO and KEGG. This was conducive to a comprehensive understanding of the biological processes and molecular pathway involved in the targets, enhancing the reliability of the results.

### 2.7 Molecular docking

The protein structure of the core target was obtained from the RCSB PDB database (https://www.rcsb.org/) (Goodsell and Burley, 2020). Search for the structural formulas of metabolites through the Pubchem database. PyMOL was used to dehydrate and hydrogenate the protein structure to ensure its suitability for molecular docking. Using CB-DOCK2 (https://cadd.labshare.cn/cb-dock2/php/index.php) performed molecular docking (Li Y. L. et al., 2025). The binding energy (BE) was calculated using the Vina software. BE < 0 kcal/mol was considered to form a stable structure between the compound and the target.

### 2.8 Druggability and toxicity analysis of core metabolites

Druggability analysis of core metabolites was conducted using the SwissADME database (https://swissadme.ch/) (Daina et al., 2017). The core metabolites were assessed for their absorption, distribution, metabolism, and excretion (ADME) characteristics. The ADMETlab 3.0 platform (https://admetlab3.scbdd.com/server/screening) provided comprehensive toxicity analysis for core metabolites (Li B. et al., 2025). This was of significant importance for drug safety assessment.

### 2.9 Animal experiments were conducted to verify the core targets

Six-week-old male C57BL/6J mice, with a weight of 16 ± 2 g, were acquired from the Laboratory Animal Center of Shanxi Medical University. The MASLD model (n = 6) was constructed by feeding 12 weeks with a 60% high-fat diet (HFD) (Dyets Inc., Bethlehem, United States of America, Catalog No. HF60). A normal diet was provided to the control group (Ctrl) (n = 6). The fasting blood glucose and random blood glucose of mice in each group were measured before the end of the experiment. At the end of the experiment, mice were anesthetized by intraperitoneal injection of 2,2,2-tribromoethanol (Meilunbio, China, Cat No. MB2548). The anesthetic was dissolved with tert-amyl alcohol (Sigma-Aldrich, United States, Cat No.72112) and prepared into a stock solution. Dilute with normal saline to the working concentration (20 mg/mL) when in use. Mice were injected intraperitoneally at a dose of 120 mg/kg to achieve anesthetic effect. Continuously monitor corneal reflex and pain reflex. After 15 min of induction, a laparotomy was performed. Collect liver and serum for subsequent research. Under anesthesia, cervical dislocation was performed to ensure death of the mice. All animal experiments strictly followed the ARRIVE guidelines to ensure the transparency and repeatability of the experiments. This study was approved by the Ethics Committee of the Second Hospital of Shanxi Medical University (Ethics Number: DW2025022).

### 2.10 Detection of biochemical indicators

According to the instructions of the kit, conduct the detection of serum biochemical indicators. It mainly includes serum total cholesterol (TC), triglyceride (TG), alanine aminotransferase (ALT) and aspartate aminotransferase (AST). The kit was purchased from Nanjing Jiancheng Institute of Bioengineering (Nanjing, China).

### 2.11 Hematoxylin-eosin (H&E) staining and oil red O staining

Liver specimens were prepared into 4-µm sections by paraffin embedding. HE staining was performed after xylene transparency and gradient alcohol dehydration. Liver specimens were prepared into 10-µm frozen sections after OCT embedding, and then Oil red O staining was performed.

### 2.12 Detect the mRNA expression levels of core targets in the liver

50 mg of liver tissue was taken and RNA was extracted using Trizol reagent (Beyotime, Shanghai, China). After determining the concentration, the reverse transcription kit from Takara Bio (Dalian, China) was used to synthesize cDNA. Subsequently, the amplification of the product for quantitative real-time polymerase chain reaction (qPCR) detection was carried out using the LineGene 9,600 Plus system (Bioer Technology, Hangzhou, China). The reaction steps were as follows: pre-denaturation at 95 °C for 30 s, followed by 40 cycles, including denaturation at 95 °C for 5 s, annealing at 60 °C and extension for 34 s. Record the cycle threshold (Ct) for each amplification cycle, and display the relative expression level of mRNA by calculating 2^−ΔΔCT^. Table 1 displayed the primer sequences utilized in this experiment.

**TABLE 1:** Primers sequence used in this study.

Gene	Forward primer (5′–3′)	Reverse primer (5′–3′)
β-actin	GGCTGTATTCCCCTCCATCG	CCAGTTGGTAACAATGCCATGT
PPARA	AACATCGAGTGTCGAATATGTGG	CCGAATAGTTCGCCGAAAGAA
PPARG	GGAAGACCACTCGCATTCCTT	GTAATCAGCAACCATTGGGTCA
ACE	AGGGGTAACATACCTCGGGA	CCTGCCTCTTATTGGGTGGG
HMGCR	AGTAAGCCCACAAAACGCAG	GGCCTTAGAGGTTCGCAAGT

### 2.13 Statistical analysis

MR analysis was performed using R software (version 4.4.0), with the TwoSampleMR and MR-PRESSO package. For animal experiments, two sets of statistical analyses were performed using Student’s t-test via Graphpad Prism (version 9.5). *P* < 0.05 was considered to have a significant difference in the result.

## 3 Results

### 3.1 MR analysis identified the gut microbiota that had a causal association with MASLD

**FIGURE 1:**
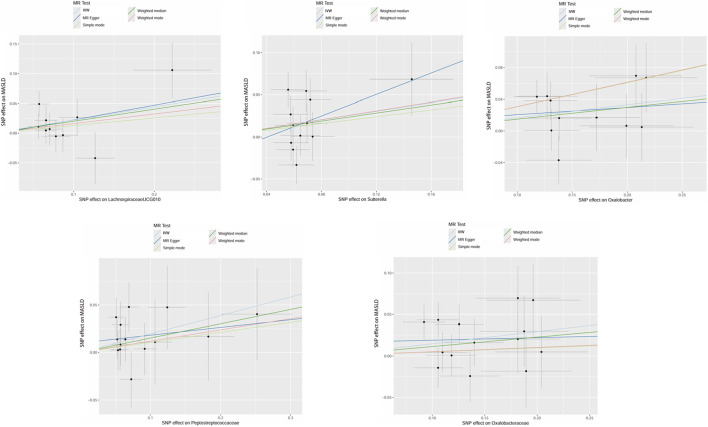
MR analysis revealed gut microbiota that could increase the risk of MASLD. Five analysis methods were adopted: IVW, MR Egger, Simple mode, Weighted Median, and Weighted Mode. Abbreviations: MR, Mendelian randomization; IVW, inverse-variance weighted.

The MR analysis results showed that 11 intestinal microbiota, including 7 genera and 4 families, had significant causal relationships with MASLD. Five of the gut microbiota were significantly positively associated with the risk of MASLD. Six gut microbiota were significantly negatively correlated with the risk of MASLD. At the genus level, we found that *Lachnospiraceae UCG010* (OR = 1.25, 95% CI = 1.02–1.52, *P* = 0.0304), *Oxalobacter* (OR = 1.18, 95% CI = 1.05–1.33, *P* = 0.0072), and *Sutterella* (OR = 1.29, 95% CI = 1.00–1.66, *P* = 0.0460) were positively associated with the risk of MASLD. Additionally, at the family level, Oxalobacteraceae (OR = 1.16, 95% CI = 1.03–1.30, *P* = 0.0112) and Peptostreptococcaceae (OR = 1.21, 95% CI = 1.02–1.45, *P* = 0.0295) also significantly increased the risk of MASLD (Figure 1).

**FIGURE 2:**
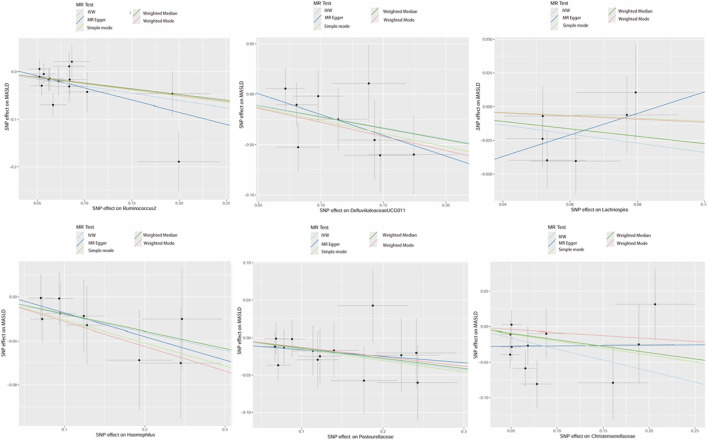
MR analysis revealed gut microbiota that could reduce the risk of MASLD. Five analytical methods were adopted: IVW, MR Egger, Simple mode, Weighted Median, and Weighted Mode. Abbreviations: MR, Mendelian randomization; IVW, inverse-variance weighted.

On the other hand, microbial groups negatively associated with MASLD risk were represented at the genus level by *Haemophilus* (OR = 0.85, 95% CI = 0.72–1.00, *P* = 0.0499), *Defluviitaleaceae UCG011* (OR = 0.80, 95% CI = 0.67–0.95, *P* = 0.0124), *Lachnospira* (OR = 0.71, 95% CI = 0.52–0.98, *P* = 0.0393), and *Ruminococcus2* (OR = 0.74, 95% CI = 0.61–0.89, *P* = 0.0013). At the family level, Christensenellaceae (OR = 0.73, 95% CI = 0.58–0.93, *P* = 0.0090) and Pasteurellaceae (OR = 0.87, 95% CI = 0.75–0.99, *P* = 0.0392) were significantly negatively associated with MASLD risk (Figure 2). As the SNPs of the two taxonomic units, Pasteurellaceae and Pasteurellales, completely overlap, the final MR results were consistent. This study prioritized the retention of lower tiers and focused on reporting the results of Pasteurellaceae.

In addition, we conducted Cochran’s Q test and found no evidence of heterogeneity. In the MR-Egger analysis, the *P* value corresponding to its intercept exhibited no statistical significance, and no significant horizontal pleiotropy signal was observed. Furthermore, the Global Test result of MR-PRESSO indicated that there was also no horizontal pleiotropy in this MR analysis. The specific results were shown in Supplementary Table S1.

### 3.2 Identify the relevant targets of microbial metabolites and MASLD

**FIGURE 3:**
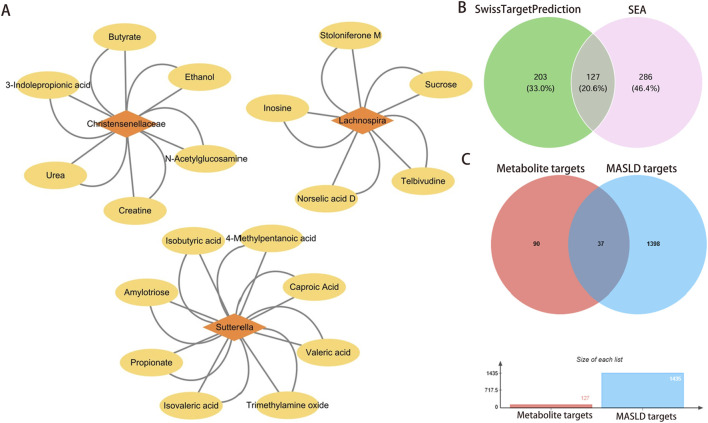
Predicting microbial metabolites and MASLD targets. **(A)** Construction of the regulatory network of gut microbiota and microbial metabolites. **(B)** SwissTargetPrediction and SEA database predicted targets of gut microbial metabolites. **(C)** The overlapping targets of gut microbial metabolites and MASLD. Abbreviations: MASLD, metabolic dysfunction-associated steatotic liver disease; SEA, similarity ensemble approach.

For the gut microbiota that had a causal association with MASLD. We used the gutMGene database to screen its related metabolites. Finally, 19 corresponding metabolites were obtained (Figure 3A). A total of 330 and 413 metabolite targets were obtained from SwissTargetPrediction database and SEA database, respectively. There were 127 common targets in the two databases (Figure 3B). Then, a total of 1,435 MASLD-related targets were retrieved from the aforementioned databases. Finally, we intersected the metabolites with the MASLD targets and identified 37 overlapping targets for subsequent analysis (Figure 3C).

### 3.3 GO and KEGG enrichment analysis of overlapping targets

**FIGURE 4:**
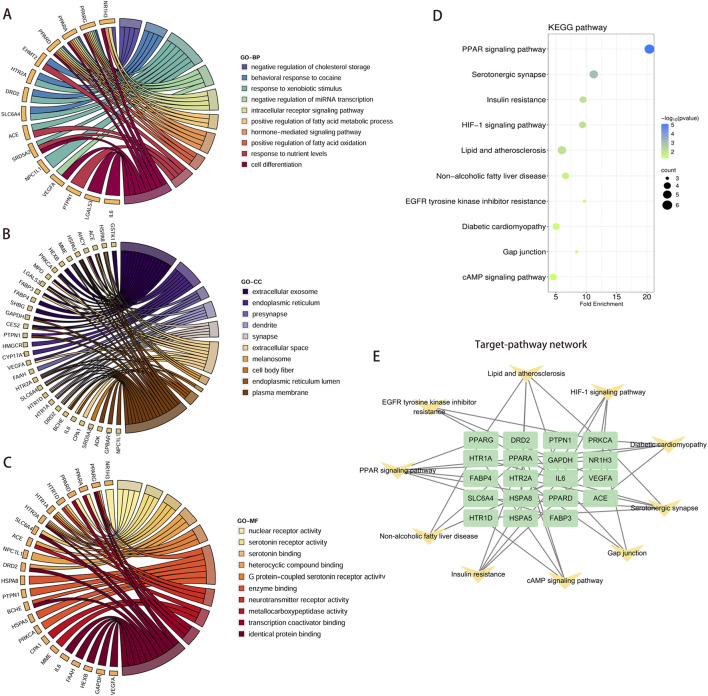
GO function enrichment and KEGG pathway analysis of overlapping targets. **(A)** The top 10 BP terms (ranked by *P* value) and corresponding genes annotated for each term. **(B)** Top 10 CC terms (ranked by *P* value) along with the target genes associated with each term. **(C)** Top 10 MF terms (ranked by *P* value) and corresponding genes annotated for each term. **(D)** Top 10 pathways identified by KEGG enrichment analysis (sorted by *P* value). **(E)** Visualization of the interaction network between KEGG pathway and targets. Abbreviations: GO, Gene Ontology; BP, Biological Process; CC, Cellular Component; MF, Molecular Function; KEGG, Kyoto Encyclopedia of Genes and Genomes.

GO enrichment analysis revealed that significantly enriched BP included negative regulation of cholesterol storage, positive regulation of fatty acid metabolic process, and positive regulation of fatty acid oxidation, etc. (Figure 4A). CC and MF analyses revealed that extracellular exosome, endoplasmic reticulum, and nuclear receptor activity were significantly enriched (Figures 4B,C). In the KEGG pathway, the peroxisome proliferator activated receptor (PPAR) signaling pathway, serotonergic synapse, insulin resistance, and non-alcoholic fatty liver disease pathways showed significant associations (Figure 4D). In addition, targeted gene-pathway network analysis revealed the potential role of genes involved in PPAR signaling and lipid metabolism (Figure 4E).

### 3.4 Construction of PPI network and core targets screening

**FIGURE 5:**
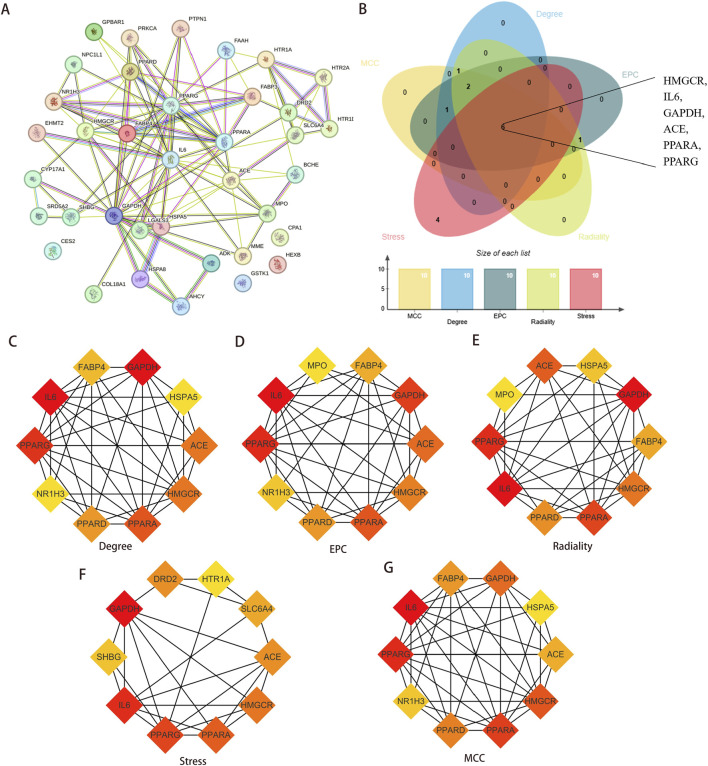
Identification of Hub genes. **(A)** Construction PPI network for overlapping targets. **(B)** The genes shared by the five algorithms were screened as Hub genes, and a total of six common targets were discovered. **(C–G)** Five algorithms were used to identify the core targets of the overlapping targets. Abbreviations: PPI, protein-protein interaction.

A PPI network was constructed for the 37 overlapping targets (Figure 5A). Hub genes were identified using five algorithms (Figures 5C–G). The six shared intersection genes—angiotensin-converting enzyme (ACE), interleukin-6 (IL6), 3-hydroxy-3-methylglutaryl-CoA reductase (HMGCR), glyceraldehyde-3-phosphate dehydrogenase (GAPDH), proliferator-activated receptor alpha (PPARA), and peroxisome proliferator-activated receptor gamma (PPARG)—were identified as core targets (Figure 5B).

### 3.5 GeneMANIA network explored the functional associations of core targets

**FIGURE 6:**
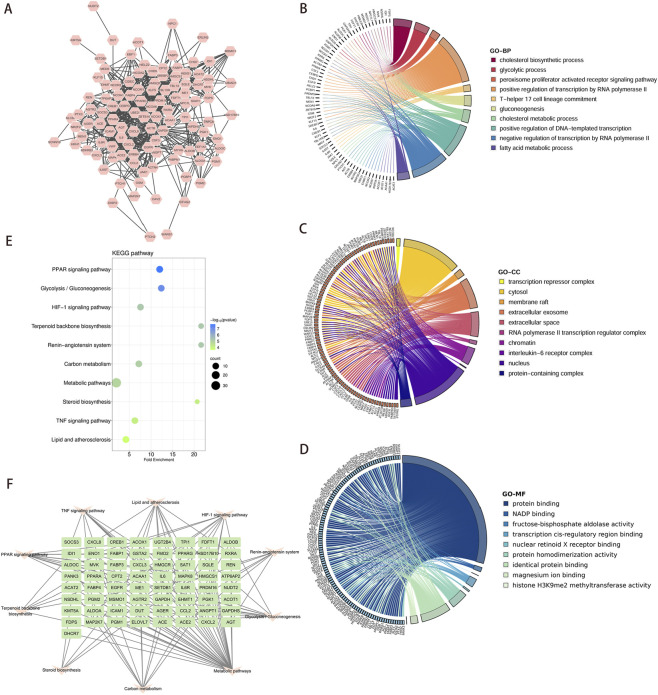
Expanding six core targets and performing functional enrichment and pathway analysis. **(A)** GeneMANIA database expands core targets to construct PPI network. **(B–D)** GO functional analysis of expanded core targets, including BP, CC, and MF. **(E,F)** KEGG pathway analysis was performed on the expanded core targets and KEGG-target network was constructed. Abbreviations: PPI, protein-protein interaction; GO, Gene Ontology; BP, Biological Process; CC, Cellular Component; MF, Molecular Function; KEGG, Kyoto Encyclopedia of Genes and Genomes.

The core gene set was expanded by incorporating the top 20 genes most relevant to the six core targets via GeneMANIA, yielding a total of 123 targets (Figure 6A). GO enrichment analysis of the 123 targets revealed that in terms of BP, the expanded gene set was primarily associated with cholesterol biosynthetic process, glycolytic process, T-helper 17 cell lineage commitment, and other processes (Figure 6B). CC indicated that these genes participated in crucial cellular structures and functions such as transcription repressor complexes, extracellular exosome, and nucleus (Figure 6C). In MF, these genes were closely associated with functions including protein binding, NADP binding, and transcription cis-regulatory region binding (Figure 6D). KEGG pathway enrichment analysis showed that the gene sets were markedly enriched in multiple metabolism-related pathways, including PPAR signaling, glycolysis/glycogen biosynthesis, HIF-1 signaling pathway and Lipid and atherosclerosis (Figure 6E). Subsequently, a gene-pathway targeting network was presented, revealing the association of these genes with multiple metabolic pathways. Especially their role in lipid metabolism (Figure 6F).

### 3.6 Compare the functional enrichment of core targets and extended gene sets

**FIGURE 7:**
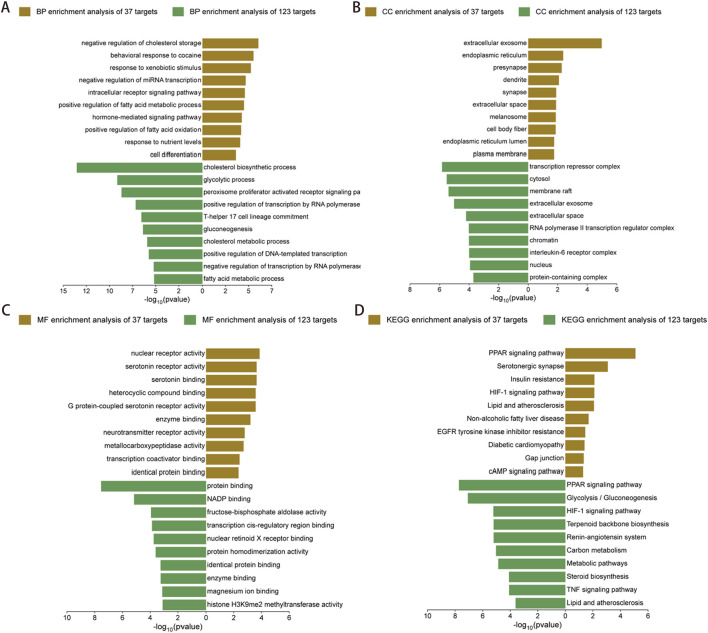
Comparative GO and KEGG enrichment analyses of 37 overlapping targets and 123 extended targets. **(A–C)** The bar chart showed the top 10 entries of 37 overlapping targets (brown bar chart) and 123 extended targets (green bar chart), including BP, CC and MF. **(D)** The top 10 KEGG pathways enriched by 37 overlapping targets (brown bar chart) and 123 extended targets (green bar chart), respectively. Abbreviations: GO, Gene Ontology; BP, Biological Process; CC, Cellular Component; MF, Molecular Function; KEGG, Kyoto Encyclopedia of Genes and Genomes.

Enrichment analysis and comparison were conducted on 37 core targets and 123 extended gene sets to identify common core pathways. In the enrichment analysis of BP, the 37 targets were mainly enriched in processes such as cholesterol synthesis, glycolysis and regulation of fatty acid metabolism. The 123 targets were significantly enriched in biological processes such as cholesterol synthesis, fatty acid metabolism and T-cell differentiation (Figure 7A). In terms of CC, 37 targets were enriched in structures such as extracellular vesicles, endoplasmic reticulum and synapses. The 123 targets focused on cellular structures such as transcription repressor complexes, cytoplasm and cell vesicles (Figure 7B). MF enrichment revealed that the core target was associated with protein binding, NADP binding and serotonin receptor activity, while the expanded gene set demonstrated significantly enriched in functions such as protein binding, transcription factor binding and homologous protein binding (Figure 7C). Through KEGG pathway enrichment analysis, it was identified that 37 targets exhibited notable enrichment within specific pathways including the PPAR signaling pathway, insulin resistance, lipids and atherosclerosis, and HIF-1 signaling pathway. The expanded gene set was enriched in important pathways such as the PPAR signaling pathway, glycolysis/glycogen biosynthesis, HIF-1 signaling, and lipids and atherosclerosis (Figure 7D). Among them, the PPAR signaling pathway, lipid and atherosclerosis, and HIF-1 signaling pathway are common enrichment pathways. It was suggested that these pathways played a crucial role in the development of diseases.

### 3.7 Constructing the gut microbiota-metabolites-targets association network

**FIGURE 8:**
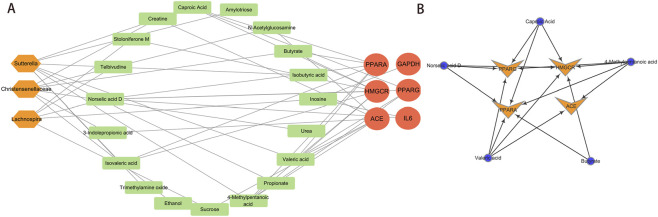
Interaction networks of gut microbes, metabolites, and key target genes. **(A)** Gut microbiota-metabolites-targets association network. **(B)** Screening core metabolites and core targets in the network.

To elucidate the therapeutic role of gut microbiota metabolites in MASLD, we constructed an associative network linking gut microbiota, metabolites, and targets to reveal complex regulatory relationships (Figure 8A). Concurrently, we identified core metabolites and core targets within this network. Cytoscape software analysis screened and identified five core metabolites and four core targets (Figure 8B).

### 3.8 Molecular docking of core metabolites

**FIGURE 9:**
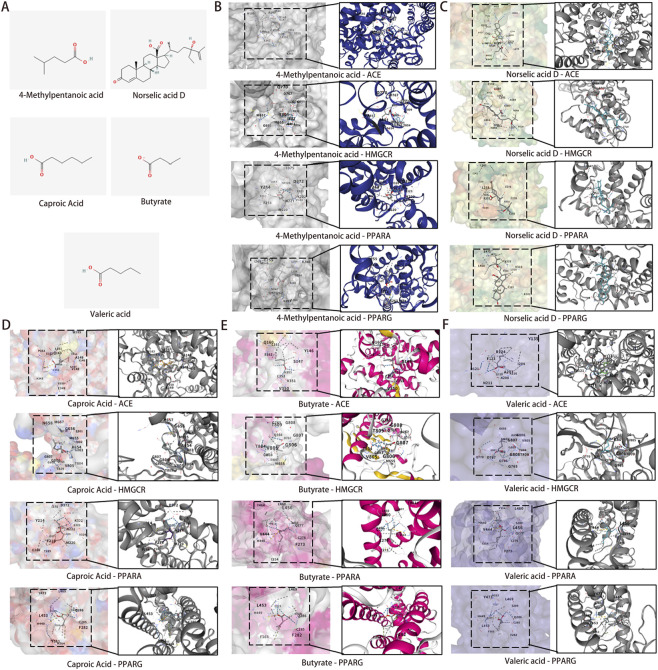
Molecular docking results of core metabolites and targets. **(A)** Two-dimensional structural diagrams of five core metabolites. **(B)** Molecular docking of 4-methylpentanoic acid with ACE, HMGCR, PPARA, and PPARG, respectively. **(C)** Molecular docking of Norselic acid D with ACE, HMGCR, PPARA, and PPARG, respectively. **(D)** Molecular docking of Caproic acid with ACE, HMGCR, PPARA, and PPARG, respectively. **(E)** Molecular docking of Butyrate with ACE, HMGCR, PPARA, and PPARG, respectively. **(F)** Molecular docking of Valeric acid with ACE, HMGCR, PPARA, and PPARG, respectively. Abbreviations: ACE, angiotensin-converting enzyme; HMGCR, 3-hydroxy-3-methylglutaryl-CoA reductase; PPARA, proliferator-activated receptor alpha; PPARG, peroxisome proliferator-activated receptor gamma.

The metabolites 4-methylpentanoic acid, Norselic acid D, caproic acid, butyrate, and valeric acid were selected as core metabolites, with their two-dimensional structural conformations displayed (Figure 9A). ACE, HMGCR, PPARA, and PPARG were selected as core targets. Molecular docking evaluated binding affinity between gut microbiota-derived essential metabolites and their targets, with BE < 0 kcal/mol indicating affinity and < −5 kcal/mol indicating strong, stable interactions. The results of molecular docking were presented in Figures 9B–F. Meanwhile, Table 2 revealed the affinity between the receptor and the ligand. Core metabolites were evaluated for drug similarity and toxicity (Tables 3, 4). All five metabolites satisfied Lipinski’s rules and key druggability criteria (molecular weight, hydrogen bond donors and acceptors, MlogP, bioavailability score >0.1, apical polar surface area (TPSA) within range). Toxicity tests revealed negative drug-induced liver injury (DILI) and Ames test results, with most metabolites showing moderate human liver toxicity (H-HT).

**TABLE 2:** Molecular docking results of core metabolites and targets.

Metabolites	Targets	Binding affinity (kcal/mol)
4-Methylpentanoic acid	ACE	−5.3
	HMGCR	−4.0
	PPARA	−4.5
	PPARG	−4.7
Norselic acid D	ACE	−8.7
	HMGCR	−7.1
	PPARA	−7.6
	PPARG	−8.2
Caproic acid	ACE	−4.6
	HMGCR	−3.9
	PPARA	−4.3
	PPARG	−4.6
Butyrate	ACE	−4.1
	HMGCR	−3.3
	PPARA	−4.2
	PPARG	−4.1
Valeric acid	ACE	−4.4
	HMGCR	−3.7
	PPARA	−4.3
	PPARG	−4.3

**TABLE 3:** Drug-likeness evaluation of core metabolites.

Metabolite	MW (g/mol)	HBA	HBD	MLOGP	Lipinski′violations	Bioavailability score	TPSA
Valeric acid	102.13	1	2	0.89	0	0.85	37.3
Caproic acid	116.16	1	2	1.27	0	0.85	37.3
4-Methylpentanoic acid	116.16	1	2	1.27	0	0.85	37.3
Butyrate	87.1	0	2	0.49	0	0.85	40.13
Norselic acid D	456.66	4	2	4.59	1	0.85	74.60

MW: molecular weight <500; HBA: hydrogen bond acceptor <10; HBD: hydrogen bond donor ≤5; MLOGP: Moriguchi octanol-water partition coefficient ≤4.15; Lipinski’sviolations≤1; Bioavailability score >0.1; TPSA: topological polar surface area <140.

**TABLE 4:** Evaluation the toxicity of core metabolites.

Metabolite	hERG	DILI	Carcinogenicity	H-HT	Ames
Valeric acid	Non-blocker	Negative	Medium	Medium	Negative
Caproic acid	Non-blocker	Negative	Medium	Medium	Negative
4-Methylpentanoic acid	Non-blocker	Negative	Medium	Medium	Negative
Butyrate	Non-blocker	Negative	Medium	Medium	Negative
Norselic acid D	Non-blocker	Negative	Positive	Medium	Negative

H-HT: human hepatotoxicity; DILI: drug induced liver injury.

### 3.9 MASLD animal model underscored the pivotal role of these core targets

**FIGURE 10:**
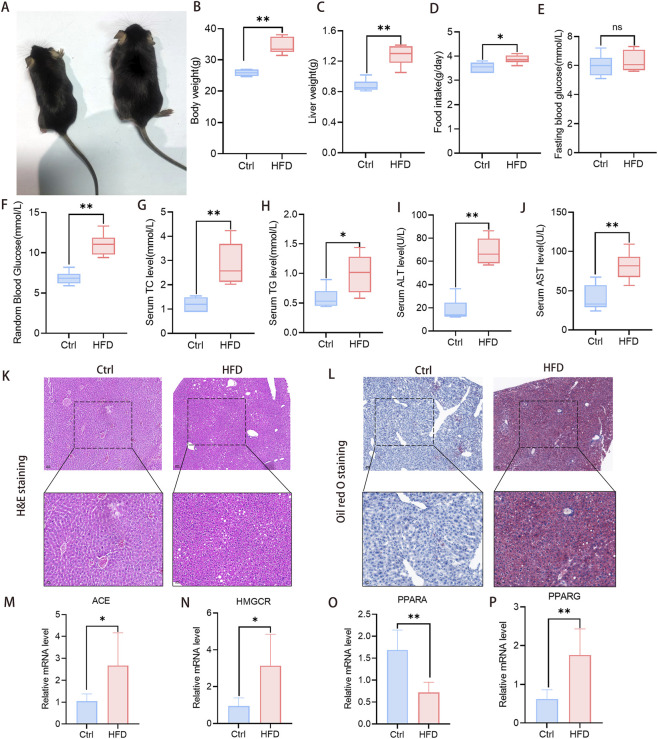
Construction of MASLD animal models demonstrating the critical role of core targets. **(A–C)** Morphology, body weight and liver weight of mice in control group and model group. **(D)** Daily food intake of mice in control group and model group. **(E,F)** Fasting blood glucose and random blood glucose levels were measured in each group of mice. **(G,H)** Changes of serum TC and TG in each group of mice. **(I,J)** The levels of serum ALT and AST in the two groups of mice. **(K)** H&E staining of the liver in control group and model group (scale bar: 50 µm and 20 µm). **(L)** Oil red O staining of the liver in control group and model group (scale bar: 50 µm and 20 µm). **(M–P)** The relative mRNA expression levels of core genes in hepatic. *p < 0.05; **p < 0.01; ns: no significant difference. Abbreviations: Ctrl, control; HFD, high-fat diet; MASLD, metabolic dysfunction-associated steatotic liver disease; TC, total cholesterol; TG, triglyceride; ALT, alanine aminotransferase; AST, aspartate aminotransferase; H&E, hematoxylin-eosin; ACE, angiotensin-converting enzyme; HMGCR, 3-hydroxy-3-methylglutaryl-CoA reductase; PPARA, proliferator-activated receptor alpha; PPARG, peroxisome proliferator-activated receptor gamma.

To verify the critical role of core targets in MASLD, we established an animal model via HFD feeding. Results showed that compared with the Ctrl group, MASLD model mice exhibited increased body weight (*P* < 0.0001), liver weight (*P* < 0.0001), and food intake (*P* = 0.0130), along with elevated random blood glucose levels (*P* < 0.0001) but no significant change in fasting blood glucose (*P* = 0.5111) (Figures 10A–F). Serum TC (*P* = 0.0013), TG (*P* = 0.0207), ALT (*P* < 0.0001), and AST (*P* = 0.0020) measurements also revealed more severe alterations in MASLD model mice (Figures 10G–J). H&E staining revealed that compared to the Ctrl group, hepatocytes in MASLD mice exhibited loose cytoplasmic staining, severe ballooning degeneration, and varying degrees of inflammatory cell infiltration (Figure 10K). Oil red O staining showed severe hepatic steatosis and lipid droplet aggregation in MASLD mice (Figure 10L). Finally, qPCR analysis demonstrated a significant upregulation in the mRNA expression of core targets ACE (*P* = 0.0273), HMGCR (*P* = 0.0129), and PPARG (*P* = 0.0032) in MASLD mice, while PPARA mRNA expression was significantly downregulated compared with control mice (*P* = 0.0009) (Figures 10M–P). These findings further confirmed the key regulatory roles of core targets in MASLD.

## 4 Discussion

MASLD is a major public health issue. Recent investigations have highlighted the crucial role of the gut microbiota to the pathogenesis of MASLD, emphasizing the necessity to comprehend the intricate relationship between gut microbiota and the disease. In this study, we used Mendelian randomization combined with network pharmacology to reveal the causal relationship between MASLD and gut microbiota. Further developed a complex regulatory network model of gut microbiota-metabolites-core targets. The key metabolites were identified for molecular docking studies, as well as for drug-like and toxicity assessments. Finally, we evaluated the expression level of the core targets in the MASLD animal model. These results provided a powerful framework for understanding the complex interactions between the gut microbiota and MASLD.

The gut-liver axis is an important bridge between gut microbiota and host metabolism (Schwabe and Greten, 2020). Dysbiosis and functional impairment of the gut microbiota may increase intestinal permeability, thereby facilitating the entry of endogenous toxins such as endotoxins into the bloodstream (Tripathi et al., 2018). These toxins can activate inflammatory responses in the liver, promoting hepatic lipid accumulation and the progression of fibrosis (Henao-Mejia et al., 2012). Our MR analysis established a causal link between gut microbiota and MASLD, highlighting the potential role of microorganisms in the pathogenesis. The accumulation of specific microbial communities could compromise the integrity of the intestinal barrier. For instance, studies have shown that *Sutterella* abundance was typically elevated in MASLD patients (Gómez-Pérez et al., 2023). Increased *Sutterella* abundance altered intestinal barrier function and promoted endotoxin release, activating hepatic immune responses and exacerbating liver inflammation (Henao-Mejia et al., 2012). Our MR analysis similarly revealed that *Sutterella* significantly increases the risk of developing MASLD. Furthermore, the gut microbiota exerted an influence on MASLD progression by affecting host energy metabolism and fatty acid metabolism (Bäckhed et al., 2004). Dysbiosis of the gut microbiota may lead to reduced insulin sensitivity in the host, increasing fat accumulation, particularly hepatic fat deposition (Le Roy et al., 2013). Disruptions in certain gut bacterial communities, such as *Oxalobacter*, Christensenellaceae, and Pasteurellaceae, may be associated with pathological features including insulin resistance and disrupted fatty acid metabolism (Goodrich et al., 2014; Wu et al., 2020). Consequently, alterations in specific gut microbiota are closely related to the onset and progression of MASLD. These microorganisms not only influence intestinal barrier function and the synthesis of metabolic byproducts, but also engage in complex interactions with host metabolism via the gut-liver axis.

The metabolites produced by the gut microbiota have a profound impact on the metabolism and function of the liver through the gut-liver axis. Among the numerous metabolites, SCFAs (including acetic acid, propionic acid, butyric acid, and valeric acid) are the key products of the fermentation of dietary fibers by the gut microbiota (Koh et al., 2016). SCFAs possess multiple biological functions such as maintaining intestinal barrier integrity, regulating lipid metabolism and immune responses. Among them, butyric acid can affect liver metabolism by activating specific free fatty acid receptors (Zhang et al., 2025). Valproic acid can regulate the cell cycle and proliferation of liver cells, maintain the function of the intestinal barrier, and regulate fatty acid metabolism (Lau et al., 2024). In the gut microbiota-metabolites-core targets network model we constructed, the core roles of several SCFAs, including butyrate, valeric acid and caproic acid, were also identified. In addition, we also predicted the presence of a novel metabolite, Norselic acid D — a newly identified highly oxidized steroidal compound that was originally isolated from the Antarctic marine sponge *Crella* sp. Early work by Ma et al. confirmed that this compound carries strong anti-infective and antiparasitic activities (Ma et al., 2009). In recent years, it has become clear that certain novel steroid compounds hold potential therapeutic value for MASLD. A well-documented instance is nor-ursodeoxycholic acid, which improves MASLD pathological progression through its antifibrotic, anti-inflammatory, and antilipotoxic effects (Halilbasic et al., 2017). Significantly, Norselic acid D shares the core steroidal backbone with these bioactive compounds, a structural feature that is closely tied to the regulation of hepatic lipid metabolism and inflammatory responses. Thus, Norselic acid D holds potential exploratory value for MASLD research, which warrants further experimental validation in subsequent studies.

Lipid metabolism plays a central role in the occurrence of MASLD. Under normal physiological conditions, the lipid uptake, synthesis and breakdown in the liver maintain a dynamic balance. In the context of MASLD, this balance is disrupted, leading to excessive accumulation of TG and free fatty acids and triggering liver lipid toxicity (Habibullah et al., 2024). Through GO and KEGG enrichment analyses, we found that the majority of metabolite targets were enriched in lipid metabolism-related pathways, including cholesterol biosynthesis and the PPAR signaling pathway, etc. The four core targets identified in this study include PPARA, PPARG, ACE and HMGCR, most of which are associated with lipid metabolism. Molecular docking analysis revealed that metabolites and core targets have strong theoretical binding potential. This suggests that these metabolites may participate in the MASLD process by interacting with the corresponding proteins. Existing literature has reported that this provides strong support for the current virtual screening results. Specifically, butyrate can activate PPARA signaling pathway in hepatocytes and block sterol regulatory element binding protein 2 signaling pathway, thereby inhibiting HMGCR expression and ultimately reducing liver lipid accumulation (Ding et al., 2024; Bridgeman et al., 2022). Valproic acid, as an inhibitor of histone deacetylase, has been proven to be able to regulate the activities of PPARA, PPARG and HMGCR (Kumari et al., 2024). We also predicted the core target ACE. Recent studies have shown that metabolites produced by the gut microbiota can effectively alleviate liver fibrosis associated with MASLD by interfering with the activity of ACE (Gao et al., 2024; Huang et al., 2010). It is worth noting that the interaction of the new metabolite Norselic acid D with the aforementioned core targets has not been reported before, suggesting that it may represent a potential regulatory pathway worthy of further exploration in the field of MASLD. It should be emphasized that the binding effect of these targets is only a predictive conclusion, and subsequent verification experiments still need to be carried out. In addition, the drug-like and toxicity prediction analysis showed that the core metabolites screened in this study have good potential for drug development. This finding further suggests that they may become potential candidate preparations for the treatment of MASLD.

However, this study still has several limitations that need to be addressed in future research. Firstly, the screening of gut microbiota metabolites in this study was completed solely based on predictive analysis. Future research should still utilize metabolomics techniques to investigate the role of these metabolites in the occurrence of MASLD. Secondly, the sample of animals in this study was relatively small. In subsequent confirmatory studies, we will expand the sample size and determine the optimal number of animals via prospective power analysis, aiming to provide more convincing experimental evidence. Thirdly, the prediction of metabolite targets relied on the SwissTargetPrediction and SEA databases. Both tools are based on the core assumption that ligands with similar structures may bind to similar targets, and their prediction outcomes are highly dependent on the ligand-target interaction data already included in existing databases. Fourthly, the molecular docking between core metabolites and targets is only a theoretical prediction. Further clarification of the biological significance of their binding needs to be achieved through experiments. For instance, *in vitro*, surface plasmon resonance and isothermal titration calorimetry can be used to verify the specificity of the binding. *In vivo*, MASLD animal models with overexpression or knockout of the target gene are constructed to detect the activity of the signaling pathways after metabolites intervention.

## 5 Conclusion

This study provided a new perspective on the role of the gut microbiota in regulating MASLD. It has revealed the close relationship between the gut microbiota, microbial metabolites and core targets in the occurrence and progression of MASLD.

## Data Availability

Publicly available datasets were analyzed in this study. This data can be found here: This study analyzed data using publicly available databases including IEU Open GWAS database (https://gas.mrcieu.ac.uk/), MiBioGen databases (https://mibiogen.gcc.rug.nl/menu/main/home/) and gutMGene database (https://bio-computing.hrbmu.edu.cn/gutmgene/#/Home).
