# Supplementary material for: Exploring the gut microbiota-microbial metabolites-targets regulatory network in metabolic dysfunction-associated steatotic liver disease
Source: Front Mol Biosci. 2026 Feb 9;13:1764479. doi: 10.3389/fmolb.2026.1764479 (PMC12926106; doi:10.3389/fmolb.2026.1764479)
Supplement: Supplementary file 1 [file Table1.docx]

Supplementary Table S1 The results of heterogeneity and pleiotropy test between the gut microbiota and MASLD in MR analysis

| **Exposure** | **Heterogeneity test** | | **Pleiotropy test** | |
| --- | --- | --- | --- | --- |
|  | **Cochran’s Q test** | **Rucker’s Q test** | **Egger intercept** | **MR-PRESSO** |
|  | **IVW *P* value** | **MR-Egger *P* value** | **MR-Egger *P* value** | **Global Test *P* value** |
| Christensenellaceae | 0.263 | 0.369 | 0.158 | 0.272 |
| Oxalobacteraceae | 0.545 | 0.486 | 0.608 | 0.396 |
| Pasteurellaceae | 0.976 | 0.964 | 0.697 | 0.747 |
| Peptostreptococcaceae | 0.891 | 0.871 | 0.532 | 0.809 |
| DefluviitaleaceaeUCG011 | 0.598 | 0.654 | 0.533 | 0.666 |
| LachnospiraceaeUCG010 | 0.466 | 0.370 | 0.920 | 0.272 |
| Lachnospira | 0.372 | 0.557 | 0.306 | 0.622 |
| Haemophilus | 0.993 | 0.987 | 0.774 | 0.990 |
| Oxalobacter | 0.517 | 0.429 | 0.793 | 0.628 |
| Ruminococcus2 | 0.489 | 0.466 | 0.415 | 0.512 |
| Sutterella | 0.115 | 0.096 | 0.545 | 0.149 |
